# Restoration of Immune Surveillance in Prostate Cancer Prevention by Sulforaphane in Hi‐Myc Mice

**DOI:** 10.1002/mc.70106

**Published:** 2026-03-09

**Authors:** Krishna B. Singh, Eun‐Ryeong Hahm, Joshi J. Alumkal, Shivendra V. Singh

**Affiliations:** ^1^ Department of Pharmacology and Chemical Biology University of Pittsburgh School of Medicine Pittsburgh Pennsylvania USA; ^2^ Department of Internal Medicine, Rogel Cancer Center University of Michigan Medical School Ann Arbor Michigan USA; ^3^ UPMC Hillman Cancer Center University of Pittsburgh Pittsburgh Pennsylvania USA

**Keywords:** immunity, prevention, prostate cancer, sulforaphane

## Abstract

Oral administration of broccoli constituent sulforaphane (SFN) prevents prostate cancer development in preclinical mouse models. However, the mechanism(s) underlying prostate cancer prevention by SFN are not fully understood. In this study, we used a human relevant mouse model (Hi‐Myc mice) to demonstrate restoration of immune surveillance by oral SFN administration. Treatment of Hi‐Myc mice with SFN for 16 weeks resulted in about 1.33‐fold increase in the number of prostate tumor‐infiltrating CD8α + T cells (*p* = 0.02 by Student's *t*‐test). The number of CD4+ helper T cells was not affected by SFN treatment. The number of CD11c/MHCII+ dendritic cells was increased by about 57% upon SFN administration. On the other hand, the number of NKp46+ natural killer cells was not significantly affected by SFN treatment. Oral administration of SFN resulted in about 30% decrease in the number of Gr1/CD11b+ myeloid‐derived suppressor cells in the prostate tumor when compared to control mice. Plasma levels of interleukin (IL)−1α, IL‐1β, IL‐4, IL‐5, IL‐10, and C‐X‐C motif chemokine ligand 2 (CXCL2 or MIP‐2) were statistically significantly lower in SFN‐treated mice when compared to control mice. Treatment of recurrent prostate cancer patients with 200 μmol/day of SFN‐rich broccoli sprout extract for 20 weeks also caused a statistically significant decrease in plasma levels of IL‐1β, IL‐4, and IL‐13. Cell proliferation inhibition by SFN in vitro was partially but significantly attenuated by IL‐4 and IL‐13 supplementation in 22Rv1 cells. These results indicate restoration of immune surveillance by oral SFN treatment in Hi‐Myc mouse model.

**Abbreviations**
BSE: broccoli sprout extract
DC: dendritic cells
IFN γ: interferon γ
IL: interleukin
MDSC: myeloid‐derived suppressor cells
MIP2: macrophage inflammatory protein 2
NK: natural killer
PBS: phosphate‐buffered saline
SFN: sulforaphane
TNF‐α: tumor necrosis factor α
TRAMP: Transgenic Adenocarcinoma of Mouse Prostate

## 1 Introduction

Sulforaphane (SFN) derived from broccoli is a promising phytochemical for prevention of prostate cancer, which continues to be a leading cause of cancer‐related deaths in American men [1, 2]. SFN is stored in plant cells as a precursor glucoraphanin, a glucosinolate [3]. SFN precursor is also present in other cruciferous vegetables like cabbage, cauliflower, kale, and so forth. Cutting or chewing of these vegetables releases myrosinase, which is responsible for the conversion of glucoraphanin to SFN, but gut microbiota can also facilitate this biochemical reaction [3, 4]. Chiao et al. were the first to demonstrate dose‐dependent apoptosis induction and growth arrest by SFN and its metabolite *N‐*acetylcysteine conjugate in an androgen‐dependent (LNCaP) human prostate cancer cell line [5]. Proliferation of androgen‐independent human prostate cancer cell line PC‐3 was inhibited significantly by SFN at media concentration of 0.02 mmol/L of this phytochemical [6]. At the cellular level, SFN can trigger apoptosis induction, G_2_/M phase and mitotic phase cell cycle arrest, autophagy induction, inhibition of cancer stem‐like cells, inhibition of metabolism and protein synthesis, induction of Phase 2 drug metabolizing enzymes, and inhibition of cell migration and invasion in human prostate cancer cells [7, 8, 9, 10, 11, 12, 13, 14, 15, 16].

At the molecular level, various transcription factors, kinases, and histone deacetylase are implicated in above discussed anticancer effects of SFN [17]. The G_2_/M phase cell cycle arrest in prostate cancer cells upon SFN treatment was attributable to checkpoint kinase 2‐mediated phosphorylation of cell division cycle 25 C phosphatase [8]. SFN‐induced apoptosis in prostate cancer cells was initiated by generation of reactive oxygen species and mediated by activation of caspases [7, 18]. SFN‐mediated apoptosis was regulated by induction of Bax and downregulation of Bcl‐2 [7, 18]. Other regulators of SFN‐induced apoptosis in human prostate cancer cells include Bak, inhibitors of apoptosis family proteins, and lysosome‐associated membrane protein 2 [7, 19, 20]. Oncogenic transcription factors inhibited by SFN include androgen receptor, signal transducer and activator of transcription 3, and nuclear factor‐κB [16, 21, 22, 23].

Mouse studies have established safety and in vivo preventive and therapeutic efficacy of SFN [18, 24, 25, 26]. Oral administration of 5.6 µmol SFN (3 times/week) inhibited growth of PC‐3 xenografts in nude mice by about 50% without weight loss [18]. Another group of investigators confirmed our finding of PC‐3 xenograft growth inhibition by SFN [24]. In Transgenic Adenocarcinoma of the Mouse Prostate (TRAMP) transgenic mice, incidence of prostatic intraepithelial neoplasia and well‐differentiated carcinoma was decreased by about 23%–28% in the dorsolateral prostate of SFN‐treated mice when compared to control mice [25]. Pharmacological inhibition of autophagy augmented prevention of prostate cancer by oral SFN administration in TRAMP mice [26]. Prevention of prostate cancer by dietary feeding of SFN‐rich broccoli sprout extract (SFN‐BSE) was also demonstrated in the TRAMP model [27].

We have shown previously that prostate cancer prevention by SFN administration in TRAMP mice is associated with suppression of fatty acid synthesis [13]. Because lipid metabolism can influence immune landscape [28], the present study was undertaken to determine the effect of oral SFN administration on prostate tumor infiltration of immune cells using Hi‐Myc transgenic mouse model of prostate cancer.

## 2 Materials and Methods

### 2.1 Reagents

SFN with purity of ≥ 98% was purchased from LKT Laboratories (St. Paul, MN). Cell culture reagents were purchased from Life Technologies ThermoFisher Scientific (Waltham, MA) or Corning (Manassas, VA). Antibodies against CD4 (cat. # sc‐19641; RRID:AB_627055) and CD8α (cat. # sc‐7970; RRID:AB_627208) were purchased from Santa Cruz Biotechnology (Dallas, TX). An antibody against perforin (cat. # ab16074; RRID:AB_302236) was from Abcam (Cambridge, UK). Anti‐granzyme B antibody (cat. # LS‐B7602) was from LSBio (Seattle, WA). Anti‐Gr1 (cat. # 108417; RRID:AB_389309), anti‐CD11b (cat. # 101208; RRID:AB_312791), and anti‐CD11c (cat. # 117312; RRID:AB_389328) antibodies were purchased from Biolegend (San Diego, CA). Anti‐NKp46 antibody (cat. # AF2225; RRID:AB_355192) was from R&D Systems (Minneapolis, MN). Anti‐MHCII (cat. # NB100‐64959) antibody was from Novus Biologicals (Centennial, CO). Recombinant IL‐4 (cat. # 6507‐IL/CF) and IL‐13 (cat. # 213‐ILB/CF) proteins were purchased from R&D Systems (Minneapolis, MN).

### 2.2 Mouse Prostate Tumor Tissue Specimens From Hi‐Myc Mice

Archived plasma and prostate tumor tissues from a previous study by us were used to determine the effect of SFN on immune landscape [29]. Briefly, 10‐week‐old male Hi‐Myc mice were divided into two groups. Control mice (*n* = 21) were orally treated with 100 µL of corn oil 5 times/week for 16 weeks, whereas experimental group of mice (*n* = 19) were orally treated with 1 mg SFN/mouse in 100 µL of corn oil 5 times/week for 16 weeks. At the end of the study, mice were euthanized by carbon dioxide inhalation and blood samples and prostate tumor tissues were collected. Plasma was obtained by centrifugation at 3000 rpm for 5 min, aliquoted, and stored at −80°C. Prostate tumor tissues were fixed in 10% neutral‐buffered formalin and sectioned at 4–5 µm thickness.

### 2.3 Immunohistochemistry

Immunohistochemistry was performed as described by us previously [30]. Briefly, formalin‐fixed paraffin‐embedded prostate tumor sections were deparaffinized, rehydrated, and washed with phosphate‐buffered saline (PBS), and then antigen retrieval was performed using a citrate retrieval buffer solution (pH 6.0) for 20–30 min at 100°C. Sections were treated with blocking buffer for 1 h followed by incubation with the desired primary antibody (anti‐CD4: 1:50 dilution; anti‐CD8α: 1:150 dilution; anti‐perforin: 1:50; anti‐granzyme B: 1:200 dilution) overnight in humidified chambers at 4°C. Slides were then washed with PBS and incubated with horseradish peroxidase‐conjugated secondary antibody for 2 h at room temperature. Sections were washed with PBS and the color was developed by incubation with 3,3′‐diaminobenzidine tetrahydrochloride and counterstained with hematoxylin. For the fluorescence immunostaining, after blocking sections were incubated with the desired primary antibody (anti‐MHCII: 1:25 dilution; anti‐CD11c: 1:50 dilution; anti‐Gr1: 1: 80 dilution; anti‐CD11b: 1:50 dilution; anti‐NKp46: 1:50 dilution) overnight in humidified chambers at 4°C. Sections were washed with PBS and incubated with Alexa Fluor 488, Alexa Fluor 568, or Alexa Fluor 633 conjugated secondary antibodies for 2 h. Slides were washed with PBS and mounted in media containing 4′,6‐diamidino‐2‐phenylindole. Stained sections were examined under Nikon confocal microscope or Leica microscope equipped with DFC 450C digital camera. Quantitation of immunofluorescence was performed using NIS Elements software. For 3,3′‐diaminobenzidine tetrahydrochloride‐stained sections, the H‐score was analyzed by positive pixel count V9 using Aperio ImageScope software.

### 2.4 Plasma Cytokine Profiling

Mouse plasma cytokine profiling was done using Luminex platform. Cytokine levels in plasma from human subjects were determined using commercially available kits.

### 2.5 Clinical Specimens

Plasma specimens from a previously published clinical study of SFN‐BSE in prostate cancer patients with biochemical recurrence were used for the measurement of cytokine levels [31]. This study was conducted at the Knight Cancer Institute, Oregon Health & Science University (Joshi J. Alumkal – Principal Investigator) [31]. The study was approved by the Institutional Review Board and registered at the ClinicalTrials.gov (NCT01228084).

### 2.6 Cell Line

22Rv1 cell line (RRID: CVCL_1045) was purchased from the American Type Culture Collection (Manassas, VA) and cultured as recommended by the supplier. The cell line was authenticated by us in March of 2017 by IDEXX Laboratories. Cells from authenticated stocks were used for the experiments conducted for this study.

### 2.7 Cell Viability Assay

The effects of SFN in the absence or presence of recombinant IL‐4 (30 ng/mL) and IL‐13 (30 ng/mL) on cell viability were determined by trypan blue dye exclusion assay as described previously by us previously [32]. Briefly, 22Rv1 cells were seeded in 12‐well plates and allowed to attach overnight incubation. The cells were then treated with the 0, 5, or 10 µM SFN in the absence or presence of IL‐4 or IL‐13 for 24, 48, and 72 h. Cells were trypsinized and stained with trypan blue. The live cells were counted under an inverted microscope.

### 2.8 Statistical Analyses

Statistical significance of difference was determined by Student's *t*‐test or one‐way analysis of variance (ANOVA) followed by Bonferroni's multiple comparison test using GraphPad Prism (version 8.0.0). A *p* value of 0.05 or lower was considered statistically significant.

## 3 Results

### 3.1 Oral Administration of SFN Increased Prostate Tumor Infiltration of CD8α + T Cells

**Figure 1:**
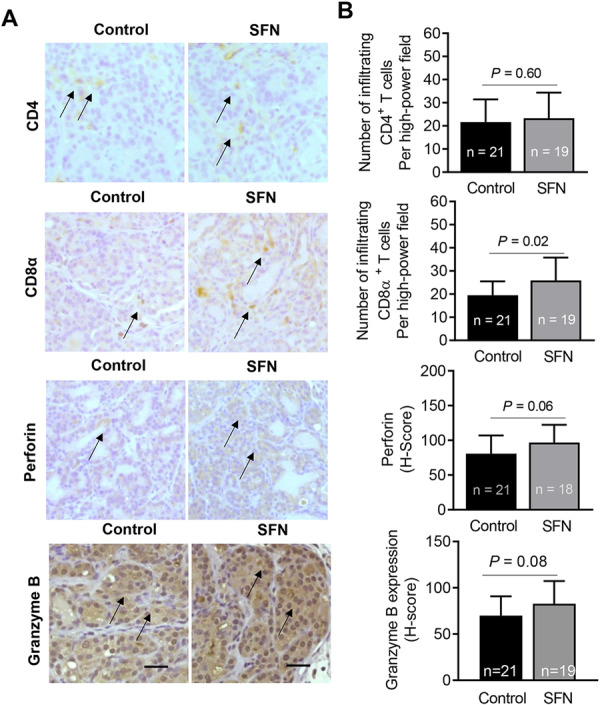
Sulforaphane (SFN) increases infiltration of CD8α + T cells in the prostate tumors of Hi‐Myc mice. (A) Immunohistochemistry for CD4, CD8α, perforin, and granzyme B in prostate tumors of control (*n* = 21) and SFN‐treated (*n* = 19) Hi‐Myc mice (20× objective magnification, scale bar = 100 µm). Arrows identify cells with positive staining for CD4, CD8α, perforin, and granzyme B. (B) Quantification of CD4 and CD8α infiltration, and expression of perforin and granzyme B proteins in the prostate tumor tissues of control and SFN‐treated mice. The results shown are mean ± SD. Statistical significance was determined by Student's *t*‐test.

Oral administration of 1 mg SFN/mouse (5 times/week) inhibited prostate adenocarcinoma burden by about 61% without causing weight loss [29]. Prostate tumor tissues from this study were used to determine the effect of SFN administration on immune landscape. The number of CD4+ helper T cells in the prostate tumor of SFN‐treated mice was comparable to that of vehicle‐treated control mice (Figures 1A and 1B). On the other hand, the number of CD8α + T cells, which are one of the important immune cells in the tumor microenvironment [33], was higher by about 33% in the prostate tumor of SFN‐treated mice when compared to control with a *p* value of 0.02 by Student's *t*‐test (Figure 1B). Cytotoxic effect of T cells is mediated by perforin, which creates pores in the target cell membrane, and granzymes (proteases) that initiate caspase‐dependent apoptosis [34]. There was a trend for an increase in expression of perforin (84%; *p* = 0.06 by Student's *t*‐test) in the prostate tumor of SFN‐treated mice when compared to control but the difference was not statistically significant due to large data scatter (Figure 1B). Expression of granzyme B protein did not differ significantly between control and SFN treatment groups (Figure 1B). These results indicated that oral administration of SFN increased prostate tumor infiltration of CD8α + T cells.

### 3.2 Oral Administration of SFN Increased Prostate Tumor Infiltration of Dendritic Cells (DC)

**Figure 2:**
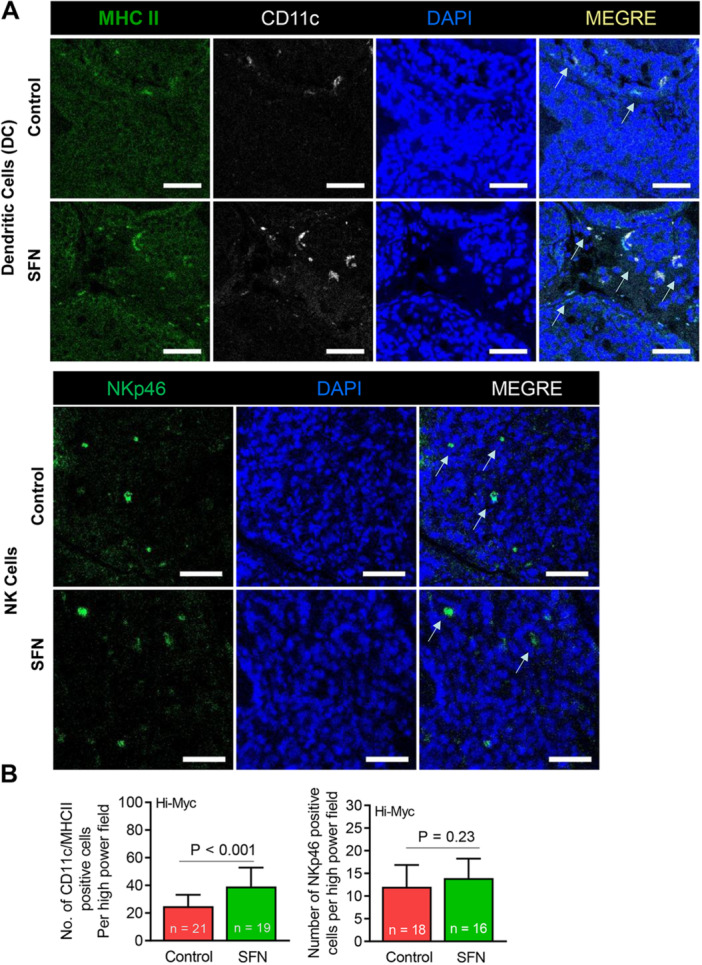
Effect of sulforaphane (SFN) administration on tumor infiltration of MHCII/CD11c+ dendritic cells and NKp46+ natural killer cells. (A) Representative immunohistochemical images of dendritic cells and natural killer cells (20× objective magnification, scale bar = 50 µm). Arrows identify dendritic and natural killer cells. (B) Quantitation of dendritic cells and natural killer cells per high‐power field in the prostate tumor sections of control and SFN‐treated Hi‐Myc mice. Results shown are mean ± SD. Statistical analysis was performed by Student's *t*‐test.

The DC regulate adaptive immunity in cancer mainly through presentation of tumor‐associated antigens, T cell priming and recruitment, and activation of tumor‐infiltrating T cells [35]. Natural killer (NK) cells are another important component of anticancer immunity [36]. The NK cells are specialized white blood cells that detect and destroy cancer cells by recognizing stress signals, creating perforation, and by releasing cytokines [36]. We determined the effect of SFN administration on DC and NK cells by immunohistochemistry for MHCII/CD11c and NKp46, respectively (Figure 2A). The number of prostate tumor‐infiltrating DC was higher by about 1.57‐fold in the SFN‐treated mice when compared to the control group (Figure 2B). On the other hand, the number of NKp46+ NK cells was comparable in the prostate tumor of control and SFN‐treated mice (Figure 2B). These results indicated that oral administration of SFN increased prostate tumor infiltration of DC without affecting NK cell infiltration.

### 3.3 Oral Administration of SFN Decreased Prostate Tumor Infiltration of Myeloid‐Derived Suppressor Cells (MDSC)

**Figure 3:**
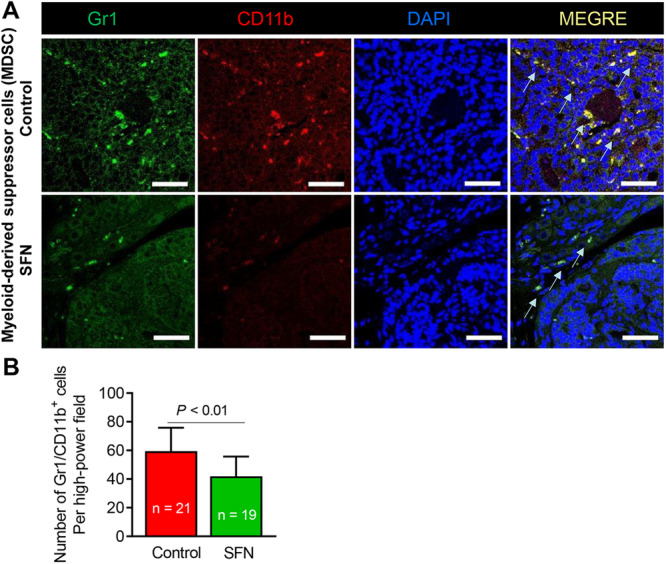
Effect of sulforaphane (SFN) administration on tumor infiltration of myeloid‐derived suppressor cells. (A) Representative immunohistochemical images of Gr1/CD11b (20× objective magnification, scale bar = 50 µm). Arrows identify myeloid‐derived suppressor cells. (B) Quantitation of myeloid‐derived suppressor cells per high‐power field in the prostate tumor sections of control and SFN‐treated Hi‐Myc mice. Results shown are mean ± SD. Statistical analysis was performed by Student's *t*‐test.

The MDSCs are a heterogeneous population of cells that accumulate in the tumor microenvironment and inhibit anticancer functions of T cells and NK cells [37]. We determined the effect of SFN treatment on MDSC population by immunohistochemical staining of Gr1 and CD11b (Figure 3A). The number of Gr1/CD11b+ MDSC was lower by about 30% in the prostate tumor of SFN‐treated mice when compared to control mice (Figure 3B). These results indicated that oral administration of SFN decreased number of MDSC in the prostate tumors of Hi‐Myc mice.

### 3.4 Oral Administration of SFN Decreased Plasma Levels of Certain Cytokines in Hi‐Myc Mice

**Figure 4:**
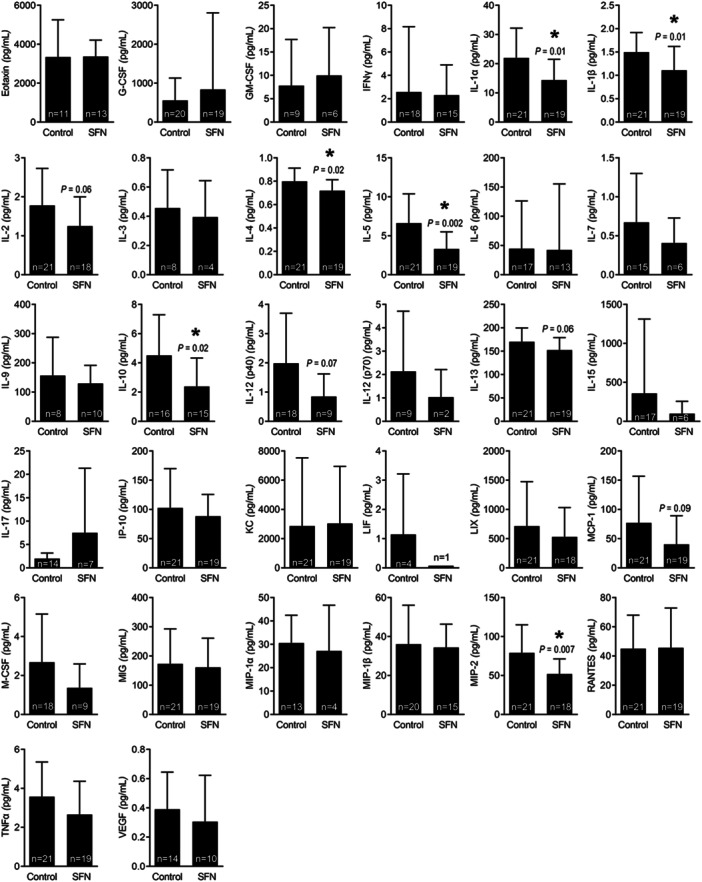
Effect of sulforaphane (SFN) administration on plasma cytokine profile in control and SFN‐treated Hi‐Myc mice. Results shown are mean ± SD. Statistical significance was determined by Student's *t*‐test.

Cytokines are utilized by immune cells to communicate with each other and with cells in the tumor microenvironment [38]. Oral administration of SFN significantly decreased plasma levels of interleukin (IL)−1α, IL‐1β, IL‐4, IL‐5, IL‐10, and macrophage inflammatory protein‐2 (MIP‐2) (also known as C‐X‐C motif ligand 2) (Figure 4). These results indicated that oral administration of SFN decreased plasma levels of certain cytokines in Hi‐Myc mice.

### 3.5 Oral Administration of SFN‐BSE Decreased Plasma Levels of Certain Cytokines in Prostate Cancer Patients

**Figure 5:**
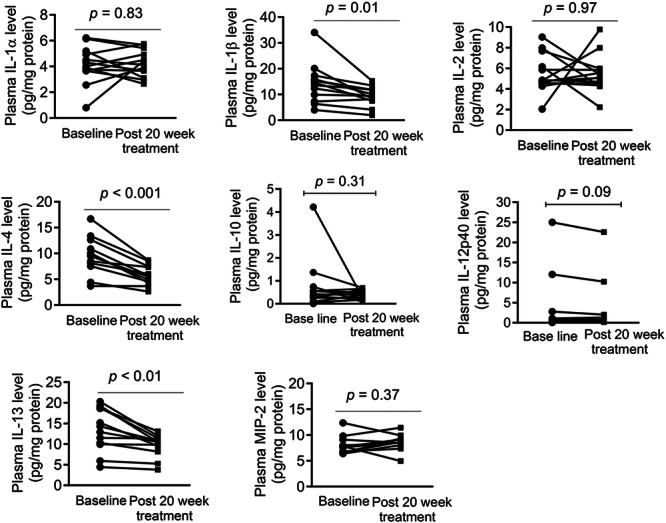
Effect of oral intake of sulforaphane‐rich broccoli sprout extract on plasma levels of cytokines at baseline (pre‐treatment) and 20‐week post‐treatment. Results shown are a before‐ and after‐treatment plot (*n* = 11–14). Statistical significance was determined by paired *t*‐test.

We measured levels of certain cytokines using plasma from prostate cancer patients with biochemical recurrence at baseline and after daily oral intake of SFN‐BSE for 20 weeks [31]. Oral SFN‐BSE administration did not significantly affect circulating levels of IL‐1α, IL‐2, IL‐10, IL‐12p40, and MIP2 (Figure 5). On the other hand, plasma levels of IL‐1β, IL‐4, and IL‐13 were reduced by about 34%, 40%, and 28%, respectively, after 20 weeks of intervention with SFN‐BSE (Figure 5). These results indicated that circulating levels of certain cytokines were decreased after daily oral intake of SFN‐BSE.

### 3.6 SFN‐Mediated Inhibition of Cell Proliferation Was Partially Attenuated by IL‐4 and IL‐13

**Figure 6:**
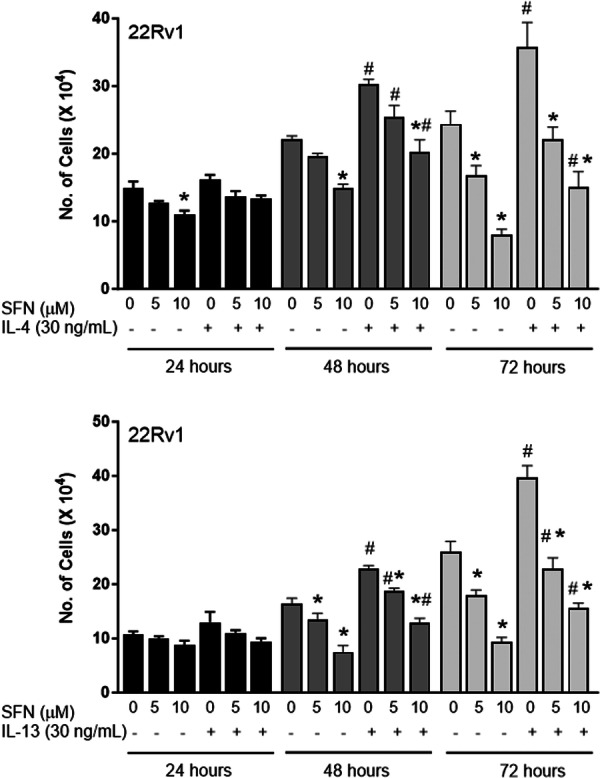
Effect of sulforaphane treatment on viability of 22Rv1 cells with or without IL‐4 or IL‐13. Results shown are mean ± SD (*n* = 3). Significantly different (*p* < 0.05). *Compared with control and #between with or without IL‐4 or IL‐13 by one‐way ANOVA followed by Bonferroni's test. Comparable results were observed in replicate independent experiments.

Figure 6 shows number of 22Rv1 cells after 24, 48, and 72 h of the culture in the absence (solvent control) or presence of SFN and/or IL‐4 or IL‐13. The SFN‐mediated proliferation of 22Rv1 was partially but statistically significantly attenuated by IL‐4 at 48‐h and 72‐h time points (Figure 6). For example, the cell proliferation by treatment with 10 μM SFN in the absence of IL‐4 was decreased by 32% and 67%, respectively (Figure 6). In the presence of IL‐4, the cell proliferation by treatment with 10 μM SFN was inhibited by 8.8% and 38.2%, respectively (Figure 6). Partial protection against cell proliferation inhibition by SFN treatment was also observed in the presence of IL‐13. These results suggest cytokine suppression by SFN may contribute to its anti‐tumor activity.

## 4 Discussion

The present study reveals that prostate cancer prevention by oral administration of SFN in Hi‐Myc mice is associated with increased prostate tumor infiltration of CD8α + T cells and DC (Figure 1). We have published previously that prostate cancer prevention by oral SFN administration in TRAMP mice was accompanied by enhanced infiltration of T cells and NK cells [25]. SFN treatment increased cytotoxicity of cocultures of NK cells and DC against TRAMP‐C1 target cells [25]. Collectively, these results indicate that in addition to growth arrest, apoptosis induction, and epigenetic modulation [7, 8, 39], restoration of immune surveillance may be an important mechanism in prostate cancer prevention by SFN. However, it is important to point out that SFN acts as a prooxidant in primary T cells leading to inhibition of its function [40].

Multiple mechanisms have been implicated in cytotoxic activity of CD8 + T cells against target cells in cancer [33]. One mechanism involves secretion of tumor necrosis factor α (TNF‐α) and interferon γ (IFNγ), which have anticancer effects in prostate cancer [41]. The circulating levels of TNF‐α and IFNγ were not decreased by SFN administration in Hi‐Myc mice (Figure 4). The second mechanism of cytotoxicity of CD8α + T cells involves production and release of cytotoxic granules [34]. The cytotoxic granules are also found in NK cells and contain two families of proteins, including perforin and granzymes [34]. Perforin protein forms a pore in the membrane of the target cells that allows the granzymes to enter the cancer cells [34]. Granzymes are serine proteases that cleave proteins inside the cells leading to apoptosis [34]. There was a trend for an increase in protein level of perforin in prostate tumors of SFN‐treated mice compared to control mice. Thus, it is possible that cytotoxic effect of CD8α + T cells in prostate cancer prevention by SFN is mediated by perforin.

The present study reveals that oral administration of SFN increases number of tumor‐infiltrating DC in the prostate tumors of Hi‐Myc mice when compared to control mice. The DC play an important role in acquired immunity and inhibit cancer progression by restoring dysfunctional immune systems leading to impediment of tumor evasion [35]. One study suggested that SFN treatment may induce an immunoregulatory response by inhibiting nuclear factor‐κB and mitogen‐activated protein kinases signaling pathways in an inflammatory environment [42]. SFN treatment modulated CD80, CD83, and B7‐H1 molecules on DC leading to T cell activation [43]. The mechanism(s) by which SFN treatment increases prostate tumor infiltration of DC in Hi‐Myc mice remain to be elucidated but several possibilities exist requiring further investigation in future studies. SFN‐mediated induction of Flt‐3 ligand, which causes expansion and mobilization of DC is one such possibility [44]. Likewise, suppression of checkpoint programed cell death protein 1 or its ligand can allow higher accumulation of DC in tumor cells [35].

Prostate cancer prevention by SFN administration in Hi‐Myc mice was accompanied by a decrease in circulating levels of several cytokines, including IL‐1α, IL‐1β, IL‐4, IL‐5, IL‐10, and so forth. The levels of IL‐1β, IL‐4, and IL‐13 were also decreased in the plasma of prostate cancer patients following 20‐week treatment with SFN‐BSE. IL‐4 and IL‐13 increased proliferation of 22Rv1 prostate cancer cells and they conferred partial protection against cell proliferation inhibition by SFN treatment. Serum levels of IL‐4 were higher in hormone‐refractory prostate cancer patients [45]. In addition, IL‐4 treatment increased PSA gene reporter activity in LNCaP cells [45]. These results suggest that IL‐4 might contribute to androgen resistance [45]. IL‐4 was shown to promote proliferation of androgen‐independent PC‐3 cells by causing activation of c‐Jun N‐terminal kinase [46]. IL‐4 was shown to increase prostate stem‐like cells by activation of STAT6 [47]. Exact role of IL‐13 in prostate cancer remains unclear, but its receptor is predictive of castration resistance [48]. Moreover, IL‐13 receptor is considered as a therapeutic target in prostate cancer [49].

In conclusion, the present study reveals that (a) oral SFN administration restores CD8α‐ and DC‐mediated immunity in the prostate tumor of Hi‐Myc mice; and (b) IL‐1β, IL‐4, and IL‐13 may serve as a biomarker of response in future clinical trials of SFN or SFN‐BSE.

## Author Contributions

Krishna B. Singh and Eun‐Ryeong Hahm were responsible for performing experiments, analyzing data, and writing first draft of the manuscript. Joshi J. Alumkal provided human plasma specimens for cytokine profiling. Shivendra V. Singh conceptualized and supervised the study and prepared final draft of the manuscript.

## Ethics Statement

Use of male transgenic FVB‐Tg (ARR2/Pbsn‐MYC) (Hi‐Myc) mice for this study was approved by the Institutional Animal Care and Use Committee of the University of Pittsburgh (IACUC Protocol # 17020271). Plasma specimens from a previously published clinical study of SFN‐BSE (NCT01228084) were used for measurement of cytokines.

## Conflicts of Interest

The authors declare no conflicts of interest.

## Data Availability

The data that support the findings of this study are available from the corresponding author upon reasonable request.
